# STAKEHOLDER ENGAGEMENT IN THE PLENARY AS A MODEL FOR PROFESSIONAL-COMMUNITY PARTNERSHIPS

**DOI:** 10.1093/geroni/igz038.2974

**Published:** 2019-11-08

**Authors:** Edward F Ansello, Sarah A Marrs, Leland H Waters

**Affiliations:** Virginia Commonwealth University, Richmond, Virginia, United States

## Abstract

The Virginia Geriatric Education Center (VGEC), a consortium of four Virginia universities, directs all initiatives in its Geriatrics Workforce Enhancement Program (GWEP) through an “all-in” interprofessional model called the Plenary. Both the structure and the process of the Plenary can serve as a model for building and maintaining successful, interdisciplinary, and intersystem partnerships that work toward shared goals. In addition to faculty and staff from the four institutions who represent nine health professions, representatives from CBOs also serve on the Plenary and attend in-person meetings twice monthly to engage in a continuous, democratic, and hands-on PDSA (Plan-Do-Study-Act) cycle to improve GWEP programs. This allows our community partners to be engaged in all components of identifying and addressing unmet needs in current and emerging interprofessional gerontology and geriatrics training, increasing CBO’s stake in the overall success of the GWEP beyond their specific involvement. Team science principles guide program improvement and growth.

